# The air was thin and my heart was open: Encouraging students to engage in environmental physiology research

**DOI:** 10.1113/EP093947

**Published:** 2026-07-14

**Authors:** Samantha L. Tsioros

**Affiliations:** ^1^ Integrative Cerebrovascular and Environmental Physiology SB Laboratory, Department of Human Health Sciences University of Guelph Guelph Ontario Canada

Lab meetings are academia's version of show and tell – you present what you have done over the past week(s) to your group and potentially receive some constructive feedback in return. When it comes time for your principal investigator (PI) to provide an update, sometimes you do not really know what you are going to get. At one of the first laboratory meetings I attended as an undergraduate student, my PI, Dr. Michael Tymko PhD, suggested that we do a patent foramen ovale (PFO) screening as a laboratory demonstration. He explained what a PFO was and why the screening was relevant to the work being done in the lab. A PFO is an opening between the heart atria, and it allows for blood to be shunted from the right to the left side of the heart; it is present in ∼30% of the population (Hagen et al., 1984). This can be more of a problem in certain pathologies, like obstructive sleep apnoea or pulmonary hypertension, when right heart pressure exceeds left heart pressure, which would cause more venous blood to be mixed with arterial blood. He then asked whether anyone would be interested in volunteering to get a screening done. Eager to get involved and curious about the physiology, I said I would do it. Our entire lab then marched down to our laboratory, where I got an intravenous catheter placed into my right arm, and another student conducted echocardiography ultrasound on my left side while lying left‐laterally. At this point I looked up and said: ‘Wait, remind me again what a PFO is!’

As I lay on the hospital bed in our laboratory, I watched another graduate student obtain a four‐chamber view of my heart. My supervisor then performed an agitated saline contrast test, also known as a bubble test, after providing some information on how it might feel and what we would see on the ultrasound screen. This test involves mixing ∼4.5 mL of saline with 0.5 mL of air between two 5 mL syringes, connected by two three‐way valves. If a PFO is present, microbubbles will appear in the right atrium and be shunted to the left atrium in ≤3 cardiac cycles (Lovering & Goodman, 2012). If a PFO is not present, all of the microbubbles will travel through the pulmonary vasculature, via the right ventricle and pulmonary artery, and be scrubbed by the alveoli. Some individuals may also have intrapulmonary shunt pathways, particularly under certain physiological conditions. These are typically identified when microbubbles appear in the left side of the heart >3 cardiac cycles after first appearing in the right side of the heart.

I think we were all expecting the screening to be unremarkable, at least I was, but then, suddenly, there were bubbles shunting across the atria… a lot of bubbles! Finding out you have a hole in your heart in front of your lab mates is not something every student can say. I heard my PI then say, ‘that is one of the larger ones I have seen’. To be completely honest, my first reaction was fear as I was concerned what this meant from a clinical standpoint. Normally, PFOs are benign; however, large PFOs can potentially be dangerous. For example, large PFOs may put you at higher risk for cryptogenic stroke, paradoxical embolism, hypoxaemia and high‐altitude pulmonary/cerebral oedema (if traveling to high altitude) (Levine et al., 1991; Seward et al., 1984; Webster et al., 1988), and it just so happened that our laboratory was preparing to go on a high‐altitude expedition to Barcroft Research Station on White Mountain, California (∼3810 m). To be on the safe side, we sent the video to a friend and collaborator, Dr. Andy Lovering, who has extensive experience with PFO‐related research, and he agreed in his email stating: ‘indeed that person has a large PFO, it would not hurt to get that checked out’. We also graded it using a traditional bubble scoring system from 0 to 5, where 0 represents no shunt is present (i.e., zero bubbles), and a score of 5 represents >12 bubbles homogeneously distributed within the left atrium. My PFO ended up being a grade 4.

I then called my family doctor, who referred me to a cardiologist, who was fascinated with the story of how I stumbled across my grade 4 PFO. The cardiologist also reviewed the video we took of my PFO screening and suggested that I get a transoesophageal echocardiograph (TEE) completed as part of some additional testing. A TEE would tell us exactly how big the hole is, and whether a PFO closure procedure would be required. While waiting for my TEE appointment, I became curious as to what it might be like to go up to high altitude with a large PFO, and whether I would have a more challenging time acclimatising compared to my peers, or whether or not I would be eligible to participate in the studies we were planning on running at the Barcroft research station. As I continued to dig through the PFO literature, I noticed that there were some high altitude‐related research questions that still had not been addressed. I began to wonder what happens to oxygen saturation during exercise and sleep at high altitude in individuals with a PFO (Lovering et al., 2011; Rexhaj et al., 2016). Previous work showed that individual with a PFO shunt ∼90 mL/min (∼1.8% of cardiac output) during rest at sea‐level (Lovering et al., 2011) and at high altitude after 16 days of acclimatization (Elliott et al., 2015). Another interesting effect is that PFO^+^ individuals have a higher resting core body temperature at rest and during exercise compared to PFO^–^ individuals. The lungs are responsible for ∼10% of total body heat dissipation, and it has been suggested that blood bypassing the pulmonary circulation through a PFO may reduce heat loss and contribute to an increase in core temperature (Davis et al., 2015). I wanted to explore this phenomenon more at high altitude. My serendipitous PFO helped me shape the perfect MSc project!

It did not take long for the cardiologist to call me to inform that I would not require a closure procedure, and that I should be fine going to the high‐altitude research station; but I might have to give up any dreams of becoming a scuba diver due to being at higher risk of decompression syndrome. This is due to air bubbles forming in the venous circulation that may travel through the PFO and enter the arterial circulation; the bubbles can become lodged in brain. I felt a sense of relief and could now focus on our laboratory's next task – White Mountain. We also submitted an amendment to our research ethics board (REB #1487) to include PFO screening in all participants, which would be part of my newly defined MSc project. We conducted our baseline testing at the University of Guelph in May/June 2025. No one in our lab (outside of Dr Tymko) had been part of a high‐altitude field expedition before, so it was difficult to imagine what the workflow was going to look like. It seemed like an impossible number of projects to complete in a short amount of time. However, we trusted the process, and with careful planning and scheduling, it went relatively smoothly; not without problems of course, but all were solvable by our team. The baseline testing at the University of Guelph felt more like a practice run for the main event at Barcroft.

I thought that the data collection experience would be similar at high altitude, but I quickly realised that was not the case. After we rapidly ascended the mountain with our robust Chrysler Pacifica, we unloaded the vans, and carrying my luggage up 15 steps to the living quarters up at Barcroft made me feel like I just completed a 100 m sprint! I have never felt breathlessness like that before. I felt like I was in the worst shape of my life. I finally understood why researchers use high altitude as a model for some disease conditions that are characterized by hypoxaemia – in short, I felt awful.

The next 24–48 h were challenging for me since I suffered from some acute mountain sickness (AMS) symptoms, which included a bad headache (∼5/10 pain), general fatigue and poor sleep quality. Thankfully, AMS, which is brought on by the rapid change in partial pressure of arterial oxygen, is usually short lived. There is some evidence that individuals with a PFO may be more susceptible to developing AMS symptoms, since they potentially have lower oxygen saturation levels (Elliott et al., 2015; West et al., 2019). However, there is some disparity within the PFO literature, and controversy over how much it meaningfully impacts physiological adaptation to hypoxia (DiMarco et al., 2022).

My AMS symptoms subsided around the 48‐h mark. I felt ready to participate in all of the exciting studies we were launching. There was one experiment in particular that I found the hardest at high altitude – the 5‐km cycling time trial. During the time trial we acquired arterial oxygen saturation by sampling blood directly from the brachial artery with simultaneous core body temperature measurements using a rectal thermistor. The study was led by a PhD student, Ms. Lauren Maier, who was a veteran with field research and had been to the Barcroft high‐altitude laboratory two times prior. I was excited to collaborate with Lauren on this project and to analyse the cardiorespiratory data she was collecting for PFO^+^ and PFO^–^ individuals. During my time trial, my arterial oxygen saturation dropped from 85.7% before exercise to 78.1% at the end. My core body temperature rose from 38°C before exercise to 38.8°C at the end of exercise. These were physiological data that I could see in real time, not only in myself, but in the other study participants. It was such an educational and exciting experience. The only downside to my MSc project was that out of the 30 participants that we screened, only six individuals (20% of our sample) presented with a PFO. However, it was an important lesson learned that sometimes field work does not go as planned. Convenience sampling is a typical limitation with field research. Although our sample size with PFO^+^ individuals is slightly lower than what others have reported, it is not unexpected. As the old saying goes, ‘sometimes you have to work with what you have’.

After 2 weeks of being at high altitude with my peers and new friends from other laboratories, our team made its way back down the mountain for celebrations in Las Vegas, Nevada. Looking back, my PFO‐focused MSc project would not have been conceived if I did not volunteer that day in the lab meeting. If I could offer any advice to current and prospective graduate students, it would be do not shy away from things that are outside of your comfort zone and take advantage of all unique opportunities presented in to you. You never know, it could lead you somewhere exciting and unexpected like it did for me. Along with a newfound passion for science, the hole in my heart has been filled with curiosity, gratitude and a deeper appreciation for the path that has led me to where I am today.

## AUTHOR CONTRIBUTIONS

Sole author.

## CONFLICT OF INTEREST

The author has no conflicts of interest.

## FUNDING INFORMATION

No funding was received for this work.

## GENERATIVE AI TOOLS

No AI tool was used in preparing this paper.
